# Exploring the synthetic biology potential of bacteriophages for engineering non-model bacteria

**DOI:** 10.1038/s41467-020-19124-x

**Published:** 2020-10-20

**Authors:** Eveline-Marie Lammens, Pablo Ivan Nikel, Rob Lavigne

**Affiliations:** 1 Department of Biosystems, Laboratory of Gene Technology, KU Leuven, Kasteelpark Arenberg 21 box 2462, 3001 Leuven, BE Belgium; 2 The Novo Nordisk Foundation Center for Biosustainability, Technical University of Denmark, Kemitorvet, Building 220, 2800 Kgs Lyngby, DK Denmark

**Keywords:** Bacteriophages, Genetic circuit engineering, Synthetic biology

## Abstract

Non-model bacteria like *Pseudomonas putida*, *Lactococcus lactis* and other species have unique and versatile metabolisms, offering unique opportunities for Synthetic Biology (SynBio). However, key genome editing and recombineering tools require optimization and large-scale multiplexing to unlock the full SynBio potential of these bacteria. In addition, the limited availability of a set of characterized, species-specific biological parts hampers the construction of reliable genetic circuitry. Mining of currently available, diverse bacteriophages could complete the SynBio toolbox, as they constitute an unexplored treasure trove for fully adapted metabolic modulators and orthogonally-functioning parts, driven by the longstanding co-evolution between phage and host.

## Abstract (web summary)

Non-model bacteria offer unique and versatile metabolisms for synthetic biology. In this Perspective, the authors explore the limited availability of well-characterised biological parts in these species and argue that bacteriophages represent a diverse trove of orthogonal parts.

## Introduction

For decades, *Escherichia coli*, *Bacillus subtilis*, *Saccharomyces cerevisiae* and, to a lesser extent, *Mycoplasma*, have been the key reference models for synthetic biology (SynBio) and the go-to biological chassis for the implementation of genetic circuits. However, advances in metabolic and genome engineering enabled the exploration of non-traditional bacterial species as chassis, including *Pseudomonas putida* and *Lactococcus lactis*, for applications that extend far beyond the traditional biotechnology and food industry^1^.

In spite of the progress, these industrially relevant organisms remain underutilized, due to the relatively limited toolboxes available for the construction of synthetic genetic circuits. The general practice of utilizing genome-editing enzymes and expression systems from the extensive *E. coli* toolbox in other hosts often leads to unreliable results, pointing to the general problem of portability in SynBio. To fully unlock the potential of non-model bacteria for SynBio, a set of standardized and reliable biological parts is urgently required, tailored to the specific requirements needed to establish genetic circuits in these organisms.

These parts could be mined from bacterial viruses (bacteriophages), as phage-based parts and enzymes are, by definition, fully adapted to the non-model host due to the longstanding co-evolution between phage and bacteria. Indeed, the study of phage–host interactions and phage biology has been a vast source of inspiration for SynBio tools, including CRISPR-Cas9-based technologies, recombineering and numerous DNA-editing enzymes^2^, but has mainly focused on mining coliphages. In this Perspective, we discuss the potential of bacteriophages to address the lack of reliable, portable tools to bridge critical knowledge and application gaps, towards fully unlocking the potential of non-traditional bacteria for SynBio applications.

## Rising stars in the field of synthetic biology

Several bacterial species have caught the attention of the SynBio community as potential biological chassis. This Perspective will focus on industrially relevant members of the *Pseudomonas, Klebsiella, Bacillus, Lactococcus*, and *Mycobacterium* genera, for which a significant number of phage genomes are available (Table 1). Moreover, these species cover different bacterial lifestyles and a wide range of SynBio applications. For instance, the Gram-negative root-colonizer *P. putida* is endowed with a highly versatile metabolism, a significant tolerance for endogenous and exogenous stresses and the capability to produce toxic, value-added compounds, including *cis,cis*-muconic acid, cinnamate, and *p*-coumarate^3,4^. Besides its natural capacity for bioremediation, *P. putida* is able to grow on structurally diverse carbon sources allowing engineered processing of abundant waste streams, such as lignocellulosic biomass and molasses^4^.

**Table 1:** Overview of non-model bacteria with high potential for SynBio applications.

	Gram-negative		Gram-positive		
Bacterial species	*Pseudomonas putida*	*Klebsiella pneumoniae*	*Bacillus subtilis*	*Lactococcus lactis*	*Mycobacterium smegmatis*
Main feature	High tolerance towards toxic compounds and stress	Glycerol fermentation to value-added compounds	Efficient protein secretion	Production of flavour- and texturizing compounds	Sterol production
Lifestyle	Aerobic	Facultative anaerobic	Facultative anaerobic	Facultative anaerobic	Aerobic
Biosafety	L1-classified	L2-classified	GRAS status	GRAS status	L1-classified
Genome reduction	Yes	Ongoing to reduce pathogenicity	Yes, but growth rates are reduced	Yes	No
Number of identified Caudovirales phages for this genus	259	71	124	133	178

Apart from *P. putida*, other Gram-negative species are on their way to become valuable biological chassis. For example, the human pathogen *Klebsiella pneumoniae* has been recognized as an outstanding glycerol fermenter for value-added, reduced compounds (e.g. diols) and research towards a safe, non-pathogenic *K. pneumoniae* chassis is currently ongoing^5^. However, not only Gram-negative bacteria are popular hosts for SynBio. Gram-positive organisms, such as *B. subtilis* and *L. lactis*, are excellent producers and in vivo delivery vehicles for therapeutic products, including oral vaccines^6^. This is due to their lack of immunogenic lipopolysaccharides and their ‘Generally Regarded As Safe’ (GRAS) status conferred by the FDA^6^. In addition, *B. subtilis* shows good growth and robustness in industrial fermentations and possesses a superior capacity of protein secretion in comparison to other hosts, whereas *L. lactis* has become indispensable in the dairy industry as a fermenter and producer of flavouring and texturizing compounds^6,7^. Furthermore, other lesser-known Gram-positive bacteria show interesting features for SynBio applications. For example, *Mycobacterium smegmatis*, which is the non-pathogenic model organism for tuberculosis research, meets all the requirements to become a valuable production host for sterols^8^ and mycobacterial proteins^9^, which often form inclusion bodies when expressed in *E. coli*.

*P. putida*, *B. subtilis*, and *L. lactis* have all been successfully submitted to chassis optimization by removing energy-draining components and other unessential or destabilizing genome loci. This has resulted in the generation of several superior strains in terms of genetic stability and physiology for heterologous gene expression^10–12^. Additionally, industrially relevant co-utilization of carbon sources and improved metabolic activity under oxygen-limited conditions have been achieved through metabolic engineering efforts^4,6,7,13^. These engineering efforts will enable broader applications of these already industrially important strains. For more information on these organisms and their applications in SynBio, we refer the reader to recent reviews^4–9^.

## Missing devices in the engineering toolbox of non-model bacteria

### Tools for large-scale genome engineering are still in their infancy

**Fig. 1:**
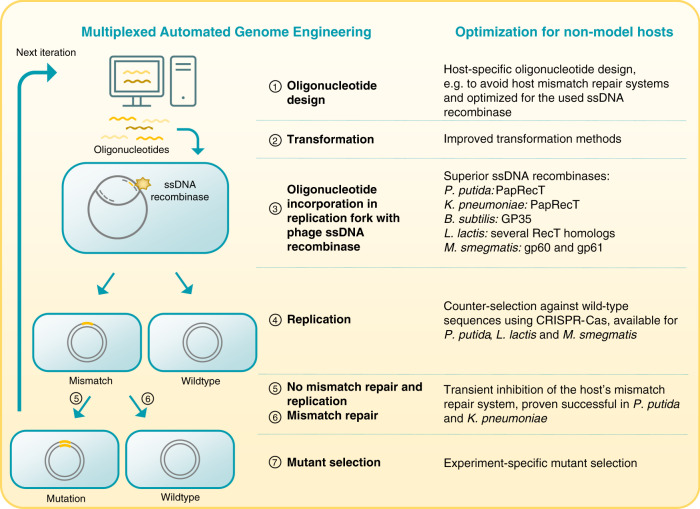
Overview of multiplexed automated genome engineering and improvement strategies for non-model hosts. 1. Oligonucleotide design should be optimized to maximally avoid the host’s mismatch repair (MMR) system and to allow optimal functioning of the used ssDNA recombinase. 2. It has been shown that poor ssDNA uptake hampers the efficiency and multiplexing of recombineering-based techniques like MAGE and DIvERGE in *P. putida*^21^, *L. lactis*^22^, and *M. smegmatis*^23^. Optimization of transformation methods is therefore essential. 3. The main focus to boost recombination frequencies of MAGE has been put on the recombinase itself, as the state-of-the-art Red-β protein for recombineering in *Enterobacteria* shows varying functionality in other hosts^153^. Superior ssDNA recombinases have been identified from prophages for *P. putida*^89^, *K. pneumoniae*^89^, *B. subtilis*^154^, *L. lactis*^22^, and *M. smegmatis*^153^, but the recombination frequencies remain well below those reported for *E. coli*, which in turn limits large-scale, multiplexed engineering efforts. Moreover, the use of alternative recombinases requires time-consuming optimization of recombinase expression levels and oligonucleotide design^21,89,153,154^. 4. CRISPR-Cas-based counter-selection of wild-type sequences has boosted the recombineering efficiency in *P. putida*^155^, *L. lactis*^156^, and *M. smegmatis*^157^. However, the drawbacks of this approach are **a** the toxicity of the Cas-induced double-strand breaks^158^, **b** the necessity of a PAM sequence near the site of mutation, and **c** the limited number of different loci that can be simultaneously edited in this setup. 5 and 6. Inhibition of the native MMR system has been shown to increase homologous recombination frequencies in multiple non-traditional hosts^21,22,159,160^. Despite its success in *E. coli*, (transient) inhibition of the MMR system to improve recombineering has not been widely implemented, probably due to the fact that mechanistic details of MMR are still largely unknown. 7. Mutant selection is an experiment-specific step for both model and non-model hosts. The RNA icon was adapted from ‘waves Icon #459771’ by Dmitry Kovalev, from the nounproject.com.

In the past two decades, the genome engineering scene has been revolutionized by ground-breaking advances in DNA-sequencing and RNA-sequencing, CRISPR-Cas9-based genome editing and innovative recombineering techniques. Metabolic and genome engineering of non-model organisms has come a long way and reliable tools for creating genetic deletions, insertions, and replacements are available^4,6,7,14^. However, large-scale reprogramming of cells is still a tedious and time-consuming undertaking, due to cycles of plasmid construction, multiple cloning steps, and inefficient plasmid curing. These problems were largely overcome in *E. coli* and related Enterobacterial species like *K. pneumoniae*, with the development of DNA recombineering and their derived, large-scale technologies including MAGE, DIvERGE, and TRMR (Box 1)^15^. While these technologies have been perfected for large-scale genome engineering of reference hosts, the upscaling of genome engineering for non-traditional microbes is trailing behind and often accompanied with unsatisfactory efficiencies. This can be explained by inadequate efficiencies of transformation methods and (counter)selection markers for non-model hosts. Moreover, the streamlined genomes of reference organisms and some key strains of *P. putida*, *B. subtilis*, and *L. lactis* allow for easy and efficient manipulation^10–12^, whereas the manipulation of alternative hosts and wild strains is hampered by native endonucleases and other interfering genomic loci. Furthermore, the specific physiology of non-enteric hosts prevents the direct transfer of *E. coli*-optimized technologies, thus requiring (often tedious and time-consuming) species-specific optimization. As an example, different strategies to boost recombination efficiencies of MAGE in non-model hosts are depicted in Fig. 1. Even with large efforts to implement and improve recombineering-based strategies in non-model hosts, these technologies are still in their infancy and further optimization is vital to allow these valuable bacterial strains to reach their full potential in the SynBio field.

Box 1 ssDNA recombineering and derived, multiplexed technologies ssDNA recombineering is a rapid and efficient method to introduce substitutions and relatively small insertions and deletions in a specific genomic locus^15^. ssDNA molecules carrying the desired mutation are electroporated into the host, after which the Beta protein of phage λ binds and anneals the ssDNA to a complementary single-strand near the replication fork. Contrary to traditional recombination methods, this method does not require constructing plasmids carrying genomic homology tails, thus shortening the genome engineering time. Moreover, this technique allows parallel and/or sequential integration of mutations by using ssDNA cocktails or multiple electroporation steps, respectively. This so-called multiplexing has led to the development of several techniques including, but not limited to, the following:

- *MAGE* or multiplexed automated genome engineering allows large-scale programming and evolution of cells by submitting the cells to several cycles of ssDNA recombineering with ssDNA cocktails carrying randomized mutations^161^. By using selective media for cell recovery, accelerated continuous evolution of the cell population can be carried out in a few days.
- CRISPR-optimized MAGE (*CRMAGE*) is a combination of CRISPR-Cas9 and MAGE to increase recombineering efficiencies^162^. The implementation of CRISPR-Cas9 allows to counterselect for wild-type sequences using targeted sgRNAs.
- Trackable multiplex recombineering (*TRMR*) is a recombineering-based method to alter gene expression levels at a genome-wide scale by introducing a strong promoter and RBS (gene overexpression) or RBS-less cassette (gene knock-down) at multiple loci^163^. The unique barcode integrated in the cassette allows to trace back the genomic alteration causing a specific host phenotype.
- *DIvERGE* or directed evolution with random genomic mutations is very similar to MAGE, but employs completely random ssDNA oligonucleotides to accelerate directed evolution^164^. This method is specifically designed to generate resistant mutants with no prior knowledge of possible resistance mechanisms.
- High-efficiency multi-site genomic editing (*HEMSE*) is a multiplexed recombineering method optimized for *P. putida*^21^. Due to lower recombination efficiencies compared to MAGE for *E. coli*, the automation aspect is not (yet) possible, hence the use of the novel HEMSE terminology.
- *ORBIT* or oligonucleotide-mediated recombineering followed by Bxb1 integrase targeting is a method for efficient genome engineering of larger genomic regions of mycobacteria in a selectable manner^23^. A Beta protein homologue allows for genomic insertion of an *attP* region. This *attP* site then acts as a landing path for a selectable cassette using the BxbA integrase to create genomic mutations, tagged genes, promoter replacements, and large insertions.

### Perfecting basic tools: learning how to walk before running

The principal requirement for efficient genome engineering of any host is a high-performance DNA transformation method, which is currently available for several non-model species, including *P. putida*^16^, *L. lactis*^17^, and *B. subtilis*^18^. In contrast, transformation of *K. pneumoniae* and *M. smegmatis* is much more difficult, as these two strains are endowed with an outer polysaccharide capsule and a thick waxy layer of mycolic acid in the cell wall, respectively^19,20^. This results in significantly reduced transformation efficiencies, in the range of only 10^4^–10^5^ transformants/µg DNA^19,20^. Since poor transformation performances will sabotage the efficacy of almost all genome-editing technologies, further optimization of transformation methods is absolutely essential. Moreover, most transformation methods have been optimized for effective uptake of plasmid DNA, whereas novel ssDNA and dsDNA recombineering techniques require efficient introduction of linear DNA. For instance, it has been shown that poor ssDNA uptake hampers the efficiency and multiplexing of recombineering-based techniques like MAGE and DIvERGE in *P. putida*^21^*, L. lactis*^22^, and *M. smegmatis*^23^ (Box 1). A valid alternative for bacterial electroporation is transduction, in which phage particles take up DNA from a donor cell and deliver it to a recipient via phage infection. Transduction has been shown to be a superior method to achieve DNA transfer in some species and wild strains, including *Mycobacterium*, for which it can mediate an almost 100% transduction efficiency^14^. However, despite this excellent efficiency, transduction is more time-consuming compared to electroporation as it requires the generation of transducing phages^14^.

Besides efficient transformation methods, markers for selection and counter-selection are indispensable in genome engineering. Antibiotic-based markers are the state-of-the-art for transformant selection and are readily available for almost all bacterial hosts. This contrasts the lack of high-performance counter-selection markers for some non-model hosts, including *P. putida* and *M. smegmatis*. For instance, the well-known levansucrase-encoding *sacB* gene from *B. subtilis* efficiently inhibits cell growth in *E. coli* and related species like *K. pneumoniae* in the presence of sucrose^24^. However, its use in other hosts sometimes requires very high sucrose concentrations (>10% w/v), thus exerting high osmotic stress and inducing spontaneous mutations in *sacB*^25,26^. To overcome this problem, novel counter-selection markers have been developed for several hosts inspired by the *URA3* and *upp* selection systems from yeast and *B. subtilis*, respectively. Although these markers show improved performance over *sacB* in *P. putida*, their utilization is strictly dependent on deletion mutants, thus limiting their application range^25,27^. To circumvent this issue, a plasmid curing system for *P. putida* based on inducible I-SceI-based plasmid restriction and conditional plasmid replication has been developed and has demonstrated to be widely applicable, shortening downtime in genome engineering^28^. This system, however, is more complex than traditional counter-selection markers.

### Urgent need for standards, norms, and regulations in synthetic biology

Apart from advances in genome and metabolic engineering techniques, the positive impact of standardization on the SynBio field should not be underestimated, as it boosts interoperability, reproducibility of results, and reduces redundant work^29^. Although the level of standardization within SynBio is still far below those of the hard sciences, large efforts led by the National Institute of Standards and Technology, the iGEM foundation and several other organizations are putting us on the right track for the future by the creation of biological part databases, software for circuit design and standards for data representation and communication^29^. However, standardized biological parts and constructs, transparent data sharing and standard operating procedures (SOPs) are largely missing for non-model species. This makes the construction of reliable genetic circuits in non-traditional microbes a process of mere trail-and-error.

The design of genetic circuitry generally starts with the selection of a suitable backbone for the construct. The development of the standard European vector architecture (SEVA) for *Pseudomonas* and other Gram-negative species constituted a pivotal point for the uniformity of vectors and standardization of biotechnological research^30^. The SEVA 3.0 database offers a wide array of replicative and integrative ‘SEVA plasmids’ following a uniform design and nomenclature, some of them endowed with expression systems that are commonly used by the *Pseudomonas* community^31^. Besides the SEVA repository, several databases are available with biological parts for both Gram-negative and Gram-positive organisms^29^, but unfortunately, dedicated parts and repositories for Gram-positive organisms remain scarce. In recent years, progress in this field has been made by developments including the iGEM Registry Probiotic Collection^32^ and the *Bacillus* BioBrick Box 2.0^33^. Since their launching, hundreds of SEVA- and *Bacillus* BioBrick vectors have been requested and the original articles have received thousands of views, indicating the success and the need for dedicated databases for non-model hosts^31,33^.

The vast majority of parts have been developed for *E. coli* and are barely validated nor characterized in other hosts. Moreover, even for this reference strain species, standardization of parts is still an illusion, as no clear consensus exists on characterization protocols for parts, let alone on standard units for the quantification of transcriptional and translational activity^29^. Therefore, biological parts cannot be reliably compared and as a consequence, researchers still tend to construct genetic circuits with parts they are familiar with rather than those possessing the optimal features to reach their goal. The absence of standard units and SOPs has not gone unnoticed and several attempts have been made to implement these in the world of SynBio for processes like fluorescent-activated cell sorting, GFP quantification, and measurements of gene expression levels^29^. Unfortunately, they have not (yet) been generally adopted by the SynBio community.

In contrast to parts standardization, methods for standard assembly of bacterial genetic constructs are much more established. The development of the restriction enzyme-based BioBrick and BglBrick standards has laid the foundation for more sophisticated assembly methods based on the Type IIs restriction enzymes^29^, including Golden Gate assembly^34^, GoldenBraid 3.0^35^, and protected oligo-nucleotide duplex-assisted cloning (PODAC)^36^. GoldenBraid and PODAC enable the generation of complex combinatorial libraries using position-specific 5′ and 3′ sequences of 4–8 nucleotides. The inherent downsides of these two techniques are the need for sequence modification to remove unwanted restriction sites and the creation of scars in between bricks, which, if reoccurring, could become hotspots for undesired recombination events^29^. Scarless and sequence-independent alternatives have been developed, including DATEL^37^ and Twin-Primer Assembly^38^, but they lack the possibility to quickly generate full or semi-random combinatorial libraries in multi-brick constructs, which is the principal strength of the aforementioned techniques. In summary, the first steps towards standardization have been taken in the field of SynBio with non-traditional hosts. To further unlock the SynBio potential of non-model microbes, there is an urgent need for a reliable, standardized, and well-characterized set of biological parts to build complex genetic circuitry.

## Constructing novel genetic circuits: the sum is more than its parts

**Fig. 2:**
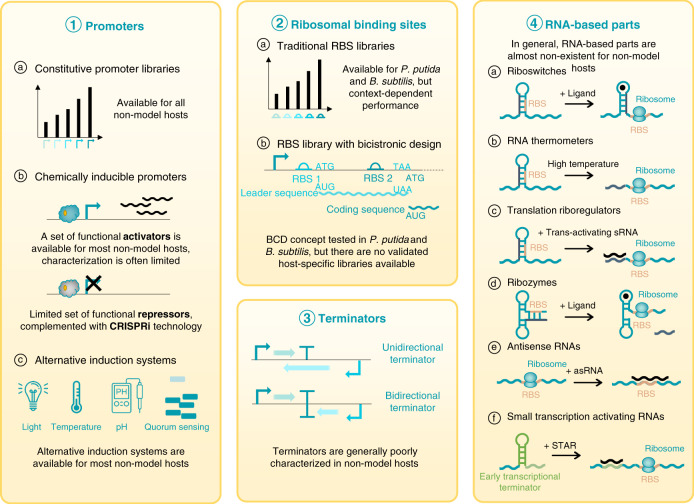
Graphical summary of different biological parts for non-model hosts. CRISPRi: CRISPR interference; RBS: ribosomal binding site; BCD: bicistronic design; sRNA: small RNA; asRNA: antisense RNA; STAR: small transcription activating RNA; The RNA, lightbulb, thermometer, and pH-meter icons were adapted from ‘waves Icon #459771’, ‘Light Icon #3393001’, ‘Thermometer Icon #342400’, and ‘ph meter Icon #3215400’ by Dmitry Kovalev, Vichanon Chaimsuk, Sarah Tan, and Vectors Point, respectively, from the nounproject.com.

As our knowledge on metabolic engineering continues to increase, we have learned that balanced expression levels and feedback mechanisms often result in higher yields and healthier cell populations^39^. To reach these desired expression levels, predictable, robust, and tuneable genetic constructs are a must and can only be built from well-characterized, reliable, and context-independent biological parts as demonstrated by Nielsen et al. ^40^. Furthermore, more complex circuits require a set of analogous high-performance parts to avoid part reuse and unwanted recombination events between them. The available set of biological parts for non-model hosts is steadily expanding, but there is still a lack of much needed well-characterized, reliable, and orthogonal parts. A graphical summary of this section is provided in Fig. 2.

### Promoters as core devices to tune gene expression

The choice for a specific promoter for both model and non-model hosts is very application-dependent, but in general, desired promoter features are predictability, tunability, independence, orthogonality, and composability^41^. Constitutive promoters are the simplest subset of promoters and are the preferred choice for industrial-scale setups, due to their high reliability and a generally low context-dependency^42^. A set of (synthetic) constitutive promoters is available for several non-model bacteria with extensive fold ranges, including *P. putida*^43^, *B. subtilis*^44^, *L. lactis*^45^, *K. pneumoniae*^46^, and *M. smegmatis*^47^.

Inducible promoters offer the user an extra level of control in terms of modulating and inducing gene expression at defined timepoints. Although many expression systems optimized for *E. coli* have been successfully transferred to other hosts, their characterization and optimization is generally limited (with a few exceptions). Due to the high context-dependent response of most expression systems, predicting protein production levels is almost impossible. Tedious optimization cycles of genetic circuit constructions are therefore required. Additionally, selecting an expression system with the optimal features for one’s goal is difficult, since each of them suffers from specific drawbacks and a centralized reporting system for expression system performance is lacking. As an example, Table 2 indicates the advantages and drawbacks of the most common expression systems used in *P. putida*. Other relevant inducible systems for *P. putida* are summarized in Supplementary Table 1 with their respective advantages and disadvantages. For other non-model hosts, the reader is referred to complementary reviews^9,44,48^.

**Table 2:** Overview and comparison of relevant induction systems often used in *P. putida*.

TF	Promoter	Inducer(s)	Reported pros, cons and main applications in *P. putida*		Ref.(s)
*Positively regulated induction systems*					
XylS	*Pm*	*m*-toluate and derivatives	+ High expression levels + Extensively characterized + Cheap inducers	− Catabolite repression − Dose-dependent response in absence of inducer metabolization pathways − Bimodal response − Leaky expression	^28,49,63,130–139^
			*Applications:* Expression of I-*sceI*, *dCas9*, *trfA*, the λ red operon, and recombinases in genome engineering tools; expression of toxic genes for controlled autolysis; production of recombinant antibody fragments and *p*-coumaric acid		
RhaRS	*P*_*rhaB*_	l-rhamnose	+ No catabolite repression + No metabolization of rhamnose: dose-dependent response + Tight regulation + Non-toxic inducer	− Inhomogeneous response at intermediate inducer levels − Expensive inducers	^132,138,140,141^
			*Applications:* Expression of dCas9 for CRISPRi and Cre for genomic deletions; production of *p*-coumaric acid		
AraC	*P*_*BAD*_	l-arabinose	+ Characterization + No catabolite repression + No metabolization of arabinose: dose-dependent response + Tight regulation + Non-toxic inducer	− Inhomogeneous response at intermediate inducer levels − Poor arabinose uptake without AraE transporter − Expensive inducers	^132,142,143^
			*Applications:* Production of *p*-coumaric acid		
*Negatively regulated induction systems*					
LacI	*Plac, PlacUV5, Ptac, Ptrc*	Lactose, isopropyl β-d-1-thiogalactopyranoside (IPTG)	+ Extensively characterized + No metabolization of lactose or IPTG: dose-dependent response	− Toxicity of IPTG − Leaky expression − Expensive inducers	^132,143–149^
			*Applications:* Expression of Bxb1 integrase for genomic integration; biodesulfurization; production of *p-*hydroxybenzoate, ethanol, and recombinant proteins		
TetR	*Ptet*	Anhydrotetracycline (aTc)	+ Tight regulation + No metabolization of inducer: dose-dependent response	− Unreliable functioning (probably due to unbalanced TetR levels) − Toxicity of aTc	^143,150–152^
			*Application:* Tubulysin production		

*TF* transcription factor.

In contrast to the relatively large set of activators, the number of (characterized) repressors for *P. putida* is low and limits the construction of complex genetic feedback mechanisms for SynBio applications (Table 2 and Supplementary Table 1). In some applications, the lack of a suitable repressor can be substituted by CRISPR interference (CRISPRi) technology^39^. This technology enables the simultaneous, tunable, and transient repression of multiple genes by expression of gene-specific sgRNAs and a single nuclease-deficient Cas9 (dCas9) and has been successfully applied in *P. putida*^49^, *M. smegmatis*^50^, *K. pneumoniae*^51^, *B. subtilis*^51^, and *L. lactis*^52^ with up to 98% repression. Although this technique offers great potential, one must note that its efficiency and efficacy remains dependent on (1) the presence of a PAM sequence within or near the promoter region for efficient transcriptional repression, (2) the strengths and weaknesses of the expression system used for dCas9 expression, (3) the burden imposed by accumulation of a large heterologous protein (dCas9), and (4) polar effects such as the repression of downstream operons.

Despite the many advantages of chemical inducers as stated above, they are often costly, toxic, and do not allow easily switching off induction. Alternative induction methods based on light, pH, temperature, and quorum sensing overcome these issues and are steadily gaining popularity in the SynBio field (Supplementary Table 1). Moreover, several of these systems are available and optimized for non-model hosts, though the number stays well below those for *E. coli*. Examples include the optogenetic CcaSR system^53,54^, the thermosensitive cI857-*P*_*L*_ system^55,56^, multiple quorum sensing-based expression systems^57,58^, and pH-regulated systems^59^.

### Ribosomal-binding sites: highly important, often overlooked

The past few years, the potential of ribosomal-binding sites (RBS) to tune expression levels has received increased attention, leading to the development of high-end RBS calculation tools^60^ and RBS libraries for some non-model hosts, including *P. putida*^21^ and *B. subtilis*^44^. However, the performance of these RBS libraries is highly sequence-dependent, due to the formation of mRNA secondary structures at the 5′ end of the transcript, which potentially inhibit translation or cause instability of the mRNA^60,61^. To overcome this problem, a library of RBS elements with a bicistronic design (BCD) was created for *E. coli*^62^. The bicistronic layout consists of two Shine–Dalgarno (SD) sites: the first SD initiates translation of a leader peptide, which in his turn holds the second SD site in its coding sequence and allows translation of the downstream gene of interest (Fig. 2 (2)). Translation of the standard leader peptide will thus disrupt secondary mRNA structures of the second SD and/or gene of interest and allows more reliable, sequence-independent translation levels than traditional RBS libraries^62^. To our knowledge, this library has not been validated in one of the aforementioned hosts, but its BCD2 element has been deployed successfully in several genetic constructs for *P. putida* and the BCD concept has been tested with promising results in *B. subtilis*^28,43,44,63^.

### Reliable transcriptional termination for qualitative genetic circuits

Proper insulation and termination of (synthetic) transcriptional units is key in genetic circuit design, as it avoids transcriptional read-through from the unit itself and interference from neighbouring promoters^40^. A set of potent terminators, and more specifically bidirectional terminators (Fig. 2 (3)), is therefore an essential part of the SynBio toolbox, leading to the development of characterized (synthetic) terminators and terminator prediction tools for *B. subtilis* and *E. coli*^64,65^. In contrast to initiation elements for which host-dependent performance has long been recognized, the set of optimized terminators for the Gram-negative and Gram-positive models *E. coli* and *B. subtilis* are often unduly considered to be generally functional in other hosts without validation. This results in poorly insulated circuits with all the undesired consequences thereof. For example, recent work demonstrated that the well-known *rrnB*-T1 terminator from *E. coli* does not fully repress gene expression in *P. putida*, despite its implementation in all SEVA vectors^31,66^. Furthermore, several *E. coli*-based algorithms were used to mine putative rho-independent terminators from environmental DNA for *Pseudomonas* species. All predicted terminators functioned in *E. coli*, but only a few showed strong termination in *Pseudomonas*, thus indicating that these tools can only be used to some extent for other hosts. Specific terminator prediction tools for non-model hosts do exist, such as GeSTer which is able to identify the typical U-trail lacking terminators of *Mycobacterium*, but they remain scarce^67^.

### RNA-based and protein-based regulation are further to be explored

Besides the classical biological parts described above, RNA-based regulatory parts allow an additional level of regulation in genetic circuits and enable the construction of elegant logic gates^68^. These RNA-based regulatory parts include riboswitches, RNA thermometers, translation riboregulators, ribozymes, antisense RNAs, and small transcription activating RNAs (STARs) with several functions (Box 2) (Fig. 2 (4)). Their fast response times and minimal cell burden compared to protein-based systems have allowed RNA-based regulatory parts to become a valuable part of the *E. coli* SynBio toolbox^68^, but to date, their use in non-model hosts is still rare.

Riboswitches and RNA thermometers are often used in SynBio to tune expression levels in response to their cognate ligand^69^. For *E. coli*, genome-wide riboswitch identification, extensive characterization, optimization, and standardization of 5′ sequences have resulted in riboswitches with reliable functioning^39,68^. In contrast, these parts are not yet exploited as regulatory SynBio tools for non-model hosts. Moreover, the first riboswitches and RNA thermometers have only recently been characterized in vivo in non-traditional bacteria^70–73^, except for *B. subtilis* for which riboswitch research is more mature^69^. The *trans*-acting counterparts of riboswitches are called translation riboregulators or antisense RNAs and can be used for multiplexed genome-wide knock-down of gene expression^39^, but their utilization as biological parts in non-traditional hosts is still limited. This might change in the near future as a synthetic sRNA toolbox for *P. putida* has recently been developed^74^.

Another set of undervalued RNA-based parts are ribozymes, whose SynBio applications include the creation of variant logic gates and detecting systems and who have demonstrated their worth as insulators of RNA parts^68^. Only a few rare examples report the use of ribozymes as tools in non-models hosts, where they have shown their utility as a glucosamine-6-phosphate responsive switch, RNA insulators and as a tool for gene inactivation in *B. subtilis, P. putida*, and *L. lactis*, respectively^75–77^. Lastly, regulation of synthetic circuits by tuning protein degradation constitutes a third regulatory level next to transcriptional and translational regulation^78^, but remains underutilized in non-model hosts. However, in contrast to RNA-based regulation, several examples are already available of controlled and/or inducible proteolysis via protein tags and orthogonal proteolysis machinery^44,66,78–81^.

To conclude, biological parts for RNA-based and protein-based regulation are highly underexploited for non-model hosts. Characterization and development of these parts (within a standardized framework) will expand the SynBio toolboxes of non-model bacteria with tools for gene expression manipulation at multiple levels.

Box 2 The diversity of RNA-based regulatory parts RNA-based regulatory parts allow an additional level of gene expression regulation complementary to the classical biological parts such as promoters and RBSs^39,68^. In general, RNA-based parts do not depend on additional proteins or translation. As such, they allow faster response times and reduced cell burden compared to protein-based systems^68^. Moreover, since these parts mostly function in *cis*, the risk of off-target effects is limited and regulator concentrations do not need to be balanced. The RNA-based regulatory parts can be divided in distinct categories (Fig. 2 (4))^68^:

- *Riboswitches* are *cis*-acting secondary RNA structures in untranslated mRNA, which control gene expression by obscuring the access to regulatory sequences like the RBS or by forming (anti)terminators in response to ligand binding.
- Similar to riboswitches are *RNA thermometers*. As the name implies, these riboswitch-like RNA structures alter their secondary structure as a function of temperature rather than ligand binding.
- *Translation riboregulators* and *antisense RNAs* are functionally equal to riboswitches, but are not part of the main mRNA transcript of a synthetic genetic construct. They regulate the construct’s expression in *cis* (encoded on the opposite strand of its target) or in *trans* (encoded in a different genomic location). Most commonly, these molecules regulate translation initiation by binding to 5′ UTR regions to block the RBS or to induce rapid mRNA degradation.
- Apart from translational regulation, sRNA-based regulation of non-translation processes is also possible, as shown by the recently developed *small transcription activating RNAs* (STARs). These small trans-acting RNAs induce transcription by disrupting an intrinsic transcription terminator upstream of the coding gene.
- A very unique set of catalytic RNA-based parts are called *ribozymes*, enzymatic RNA molecules able to catalyse biochemical reactions such as RNA cleavage and ligation. In some cases, the activity of these ribozymes is dependent on the binding of a specific ligand, which further broadens the application range of these RNA enzymes.

## Phages are unexplored troves for synthetic biology tools and parts

From the inception of molecular biology, bacteriophages have proven to be indispensable tools to conduct biomolecular research^2^. Indeed, the billion-year-long ongoing evolutionary arms-race between bacteria and phages has resulted in the emergence of phage enzymes which modify the bacterial genome and the metabolism at different levels. These phage elements reshape the bacterial cell into a highly efficient phage-particle factory in a matter of minutes^82^. Currently, mostly the exploitation of coliphages has resulted in the development of SynBio tools including phage display, Gateway cloning, recombineering and the famous pET-expression system for recombinant protein production^2^. The diversification towards phages infecting other species could potentially expand and revolutionize their respective SynBio toolboxes for genome, metabolic, and even cell engineering. More specifically, the number of identified phages infecting *Pseudomonas*, *Klebsiella*, *Bacillus*, *Lactococcus*, and *Mycobacterium* genera is steadily rising (Table 1), as such, these phage genomes can be readily explored for novel parts and tools.

### Efficient genome engineering using host-specific phage enzymes

**Fig. 3:**
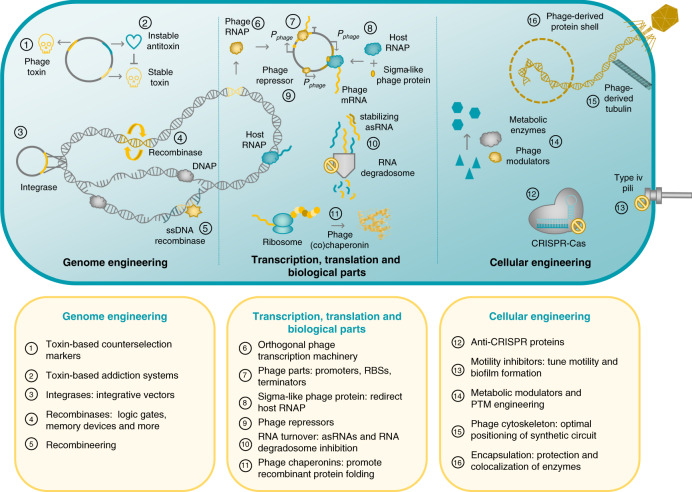
Overview of SynBio applications of phage proteins. Phage proteins show high potential for use in genome engineering (left), manipulation of transcription and translation processes (middle) and cellular engineering (right). Besides phage proteins, phage genomes are a large source of orthogonally functioning biological parts for synthetic genetic circuitry (middle). The RNA, DNA, and bacteriophage icons were adapted from ‘waves Icon #459771’, ‘DNA Icon #3383522’, and ‘bacteriophage Icon #467715’ by Dmitry Kovalev, Muhammad Tajudin, and Dong Ik Seo, respectively, from the nounproject.com.

Phage genomes encode many toxins to halt cellular growth, which have been successfully applied as treatments against bacterial infections including the engineered endolysin-based “Artilysins”^83^. Besides their obvious applications as antimicrobials, phage toxins can also be used in SynBio as (inducible) counter-selection markers for non-traditional hosts (Fig. 3 (1)). Notably, several of these toxic proteins are host-specific, such as gp8 and gp18 from *Pseudomonas* phage LUZ7, a potentially interesting feature for manipulation of expression vectors^84^. For instance, an expression vector which constitutively expresses a host-specific toxin, can be replicated easily in *E. coli*. After substituting the toxin for a gene of interest, the non-traditional expression host can be directly transformed with the recombinant vector, while the toxin counterselects against transformants carrying the unmodified vector^85^. The toxin thus allows to skip intermediate cloning steps to *E. coli* saving valuable time and effort. Apart from toxins, some phages encode antitoxins or toxin–antitoxin modules to bypass host-encoded abortive infection mechanisms^86^. These toxin–antitoxin pairs could be used to create plasmid-addiction systems or stabilize genomic inserts in situations where the use of antibiotics is undesirable or impossible, such as large bioreactors or human health applications (Fig. 3 (2)). The most well-known example of a phage-based addiction system is the *phd-doc* toxin–antitoxin module of temperate coliphage P1^87^, but toxin–antitoxin modules have also been identified in prophages in other bacteria, including *txpA/ratA*, *bsrG/sr4,* and *yonT/as-yonT* in *B. subtilis* prophage elements^88^.

The discovery of integrases and recombinases from temperate phages have revolutionized the SynBio field and resulted in a myriad of genome engineering tools^2^. At first, these enzymes were primarily used for genomic integration of synthetic circuitry or to rearrange DNA segments (Fig. 3 (3 and 4)). Soon after, their application range was expanded to include combinatorial and reversible DNA assembly methods, various logic gates, analogue-to-digital converters, memory devices, and multiplexed DNA editing via recombineering (Fig. 3 (4 and 5))^2^. To date, the use of these versatile enzymes in non-model hosts has mostly remained limited to genomic integration tools and recombineering. The characterization of existing and novel integrases and recombinases from prophages will optimize the currently available recombinase-based tools for these bacteria^89^. Additionally, they will enable the implementation of more advanced applications, including the construction of complex logic gates and memory devices, thus allowing non-model hosts to reach their full potential as valuable biological chassis for SynBio applications.

### *Autographiviridae* are a goldmine for orthogonal transcription

A specific family of phages, the *Autographiviridae*, encode their own transcriptional machinery for viral replication^90^. The archetype of this class is the well-known T7 phage, which has a small, single-subunit RNA polymerase (RNAP) and strong T7 promoters, RBSs and terminators, which have made their mark on the SynBio field^2^. Apart from the broad-use, fully orthogonal pET expression system for *E. coli*, several other T7-based applications emerged, such as AND-gates based on fragmented T7 RNAPs and a resource allocator^2,91^. So far, the use of the pET system in other hosts has been restricted by severe cytotoxicity of the T7 RNAP^92^. We argue that T7-like phages, like gh-1 and KPO1K2 infecting *P. putida* and *K. pneumoniae*, respectively^93,94^, should be explored as a source of similar orthogonal RNAPs and biological parts specifically tailored to their Gram-negative bacterial host, with reduced toxicity (Fig. 3 (6 and 7)). Beyond this family, the N4-like bacteriophages encode multiple phage RNAPs and corresponding RNAP-specific promoters, allowing a gradual shift from early to middle and late gene expression^95^. The timed aspect of this transcriptional scheme shows high potential for sequential gene expression in synthetic constructs.

In contrast to the *Autographiviridae*, other phage families are completely dependent on the host transcription machinery and use different strategies to regulate transcriptional expression. For one, *Pseudomonas* phage 14-1 and *Bacillus* phage SPO1 encode sigma factor-like proteins gp12 and gp28, respectively, to alter the RNAP’s promoter specificity towards phage-specific promoters^96,97^. These proteins, in combination with their native phage promoters, could be used to favour transcription of heterologous genes and at the same time avoid cellular stress due to overexpression by balancing the total transcriptional load, which is a common concern with the pET system (Fig. 3 (8))^91^. Secondly, temperate phages rely on transcriptional repressors for their lysogenic switch, which can be exploited for SynBio purposes like the cI and Cro repressors from coliphage λ (Fig. 3 (9))^98^. Besides repressors, also transcriptional activators have been identified in phage genomes, including ORF2 of *Lactococcus* phage phi31^99^. Thirdly, apart from transcriptional initiation, other steps in the protein production process are also prone to viral interference, which could inspire novel SynBio tools or interesting approaches to increase bioproduct yields. For example, phage mRNA translation is influenced by secondary mRNA structures, self-splicing introns, frameshifting and the remarkable translational bypassing phenomenon^100^, which could lead to novel translation regulatory tools. Programmed translational frameshifting is the process where a repeat of nucleotides allows the ribosome to slip and continue translation in a different reading frame, as observed in many dsDNA phages including *Lactococcus* phage Q54^101^. These viral frameshifting events could inspire synthetic programmed frameshift to generate signal splitters in genetic circuitry: e.g. the expression of one coding sequence results in two enzymes with different functioning^102^. The translational bypassing phenomenon has so far only been observed in coliphage T4, where the mRNA of *gp60* contains a stretch of 50 non-coding nucleotides, but is still translated into a single polypeptide. Instead of dissociating from the *gp60* mRNA when reaching the non-coding gap, the ribosome “glides” over this non-coding region and continues translation at the second coding region^103^. Further research on this phenomenon may indicate if engineered translational bypassing can be used in controlled expression of designer proteins^103^. Furthermore, RNA turnover is altered during phage infection by stabilizing viral mRNA via asRNA-masking of RNase E degradation sites or inhibition of the RNA degradosome, e.g. by Dip of *Pseudomonas* phage phiKZ^104,105^. These could potentially become interesting approaches to elevate mRNA abundance of a desired transcript and increase protein yield (Fig. 3 (10))^106^. Lastly, *Pseudomonas* phages EL and OBP and *Bacillus* phage AR9 promote protein folding with efficient phage chaperonins which do not require any additional co-factors^107–109^. These chaperonins can be co-expressed in industrial bioreactors to elevate soluble and functional levels of recombinant protein, a proven method for *E. coli*-based recombinant protein production using more complex bacterial chaperonins (Fig. 3 (11))^110^.

### Beyond traditional engineering: phage-based cellular regulation

During phage infection, phages do not only alter transcription and translation-related processes as described above. They remodel the entire cell metabolism, core processes, and cell physiology to create an optimal environment for phage replication^82^. To do so, they have acquired high-end cell modulators, optimized to their host’s specific cell physiology. These modulators constitute a goldmine of revolutionary, strain-optimized tools to expand the SynBio toolbox, as we illustrate here using selected examples. First, when entering the cell, the phage protects itself from several host defence mechanisms by using anti-CRISPR proteins (Acr) or by integrating non-canonical nucleotides into their genome to avoid host restriction enzymes^111,112^. Since the discovery of Acr proteins in *Pseudomonas* phages, many other Acr proteins have been identified and used in a range of SynBio applications, including regulation of CRISPR-Cas-based tools at multiple stages, reduction of CRISPR-Cas toxicity and the development of Acr-based CRISPRi and selection markers (Fig. 3 (12))^112^. In contrast, the DNA manipulation enzymes still need to find their way to the in vivo SynBio field, but could for example be used to protect DNA sequences from nucleases in restriction-based DNA assembly methods. Apart from the host defence mechanisms, phages also raise their own defence systems against superinfection by rivalling phages, showing interesting features for SynBio applications. For instance, phage proteins, such as Tip from *Pseudomonas* phage D3112, have been identified which inhibit type IV pili-formation, a common adsorption site among phages, thus establishing superinfection exclusion^113^. These inhibitors could allow us to transiently inhibit twitching motility and manipulate biofilm formation to optimize microbial cell-factories in a more flexible manner, an approach already proven successful for *Pseudomonas aeruginosa* fuel cells (Fig. 3 (13))^114^.

After successful cell entry, the phage hijacks the cell’s metabolism to favour viral replication. Examples of altered metabolic pathways include the phosphate and nitrogen metabolism, photosynthesis, the pentose phosphate pathway, and nucleic acid synthesis^82^. For example, *Pseudomonas* phage phiKZ promotes the de novo synthesis of pyrimidine nucleotides by reprogramming the pyrimidine pathway with seven predicted modulators^115^. These metabolic modulators could aid to overcome bottlenecks in metabolic engineering or could indicate key targets of metabolic pathways (Fig. 3 (14)). Furthermore, bacteriophages secure their energy supply for phage-particle production by shutting down non-essential energy-draining processes such as host transcription, DNA replication, and cell division^116,117^. In a similar fashion, these inhibitory proteins could be used to uncouple cellular growth from protein production to maximize substrate-to-product conversion and product yield in bioreactors, as demonstrated successfully in *E. coli*^118^. In conclusion, phage genomes are an underexploited treasure trove of metabolic modulators and cellular engineering tools, which could allow us to efficiently manipulate cells on multiple levels and revolutionize the SynBio toolbox.

## Concluding remarks and outlook

In recent years, several alternative bacterial hosts for SynBio applications have emerged alongside *E. coli*, the reference Gram-negative bacterium for such purposes. With further optimization, these hosts could be developed into an established set of biological chassis with an ideal option for every possible SynBio application. To reach this goal, perfecting current genome engineering technologies, creating biological standards and optimizing and expanding the set of biological parts, tailored to each host, are absolutely essential. Bacteriophages are not only a vast source of potential host-specific biological parts and genome engineering tools, their genomes also encode many metabolic modulators and other interesting enzymes for directed cell manipulation, which can extend and revolutionize the SynBio toolbox^2,82^. These phage genomes are becoming increasingly available and can be readily exploited for *Pseudomonas*, *Klebsiella*, *Bacillus*, *Lactococcus* and *Mycobacterium* genera (Table 1). In contrast, for other industrially relevant species (including the ultrafast-growing and upcoming host *Vibrio natriegens*^119^), the number of identified phages is still extremely low, thus limiting the SynBio potential of phages for these species for the time being.

To date, protein expression levels are mostly manipulated on the transcriptional level. However, recent research has revealed the importance of the amino acid composition of N-terminal and C-terminal protein regions, post-translational modifications (PTMs) and cellular spatial organization on protein expression, stability, and enzymatic activity^120–124^. These novel approaches to tune expression levels could soon constitute a new generation of established SynBio tools for reference bacteria. Interestingly, phages employ these strategies naturally to regulate expression levels and manipulate the cell, and could be exploited in phage-based SynBio tools for multilevel cellular engineering in the future. In contrast to regulation of transcription and translation, PTMs allow a very quick adaptation of protein function, localization, or stability to a changing environment^124^. In spite of this major advantage, the manipulation of PTMs for SynBio purposes remains limited due to the difficulty to alter specific PTMs in vivo. The use of phage kinases and acetylases could facilitate PTM engineering in the future, as such enzymes have been discovered to specifically manipulate host proteins of metabolic pathways and host defence mechanisms (Fig. 3 (14))^125,126^.

Some phages build their own cytoskeleton to centrally position their genome in the host to optimize phage-particle production^127^. To our knowledge, physical positioning of DNA as an optimization strategy for recombinant protein production is unknown territory so far, but could significantly increase protein yields in the future (Fig. 3 (15)). Furthermore, it has been proposed that jumbo phages encapsulate their genome in a nucleus-like proteinaceous shell upon cell entry to evade CRISPR-Cas^128^. Similar strategies including synthetic physical encapsulation or co-localization of enzymes via scaffold molecules have already proven effective to protect molecules from degradation by host enzymes and, moreover, to increase metabolic flux and reduce cross-talk^122,123^. By further studying phage encapsulation and shell proteins, this could lead to improved synthetic encapsulation and co-localization of enzymes (Fig. 3 (16)).

On a critical note, one potential limitation of phage-based parts and tools is that they are mostly species-specific, thus limiting portability to other species. However, recent examples indicate that some phage recombinases may display broad host specificity^89^. Rather than solving the portability issue within SynBio, we call for caution when using *E. coli*-optimized tools blindly in other hosts and promote the development of host-specific toolboxes instead. Furthermore, while several examples of potential novel tools have been proposed and discussed here, it should be obvious that we have only just scratched the surface of the potential bacteriophages hold for SynBio. Indeed, the majority of world’s bacteriophages remains unidentified and up to 40–90% of viral sequences cannot be aligned^129^. The functional potential to be found in this ‘viral dark matter’ is boundless and one can only imagine the great innovations that remain to be discovered.

## Supplementary information

Supplementary Information
