# Supplementary material for: Exploring the synthetic biology potential of bacteriophages for engineering non-model bacteria
Source: Nat Commun. 2020 Oct 20;11:5294. doi: 10.1038/s41467-020-19124-x (PMC7576135; doi:10.1038/s41467-020-19124-x)
Supplement: Supplementary file 1 — Supplementary Information [file 41467_2020_19124_MOESM1_ESM.docx]

**Supplementary Table 1: Overview and comparison of relevant induction systems often used in P. putida**. TF: transcription factor; the concentration refers to the generally used concentrations for induction in P. putida.

| **TF** | **Promoter** | **Inducer(s)** | **Concentration** | **Reported pros, cons and main applications in *P. putida*** | | **Ref(s)** |
| --- | --- | --- | --- | --- | --- | --- |
| ***Positively regulated induction systems*** | | | | | | |
| **XylR** | *Pu, Ps1* | *m*-xylene and derivatives |  | - High expression levels - Extensively characterized - Cheap inducers | - *m*-xylene is toxic and volatile - Bimodal response - Catabolite repression | ^1–6^ |
|  |  |  |  | *Application:* 2,4-DNT biosensor | |  |
| **XylS** | *Pm* | *m*-toluate and derivatives | 0.1-1 mM  (m-toluate) | - High expression levels - Extensively characterized - Cheap inducers | - Catabolite repression - Dose-dependent response in absence of inducer metabolization pathways - Bimodal response - Leaky expression | ^2,7–18^ |
|  |  |  |  | *Applications:* Expression of I-*SceI*, *dCas9*, *trfA*, the λ red operon and recombinases in genome engineering tools; expression of toxic genes for controlled autolysis; production of recombinant antibody fragments and *p*-coumaric acid | |  |
| **RhaRS** | *P_rhaB_* | L-rhamnose | 1 mM | - No catabolite repression - No metabolization of rhamnose: dose-dependent response - Tight regulation - Non-toxic inducer | - Inhomogeneous response at intermediate inducer levels - Expensive inducers | ^10,14,19,20^ |
|  |  |  |  | *Applications:* Expression of dCas9 for CRISPRi and Cre for genomic deletions;  production of *p*-coumaric acid | |  |
| **AraC** | *P_BAD_* | L-arabinose | 1 mM-10 mM | - Characterization - No catabolite repression - No metabolization of arabinose: dose-dependent response - Tight regulation - Non-toxic inducer | - Inhomogeneous response at intermediate inducer levels - Poor arabinose uptake without AraE transporter - Expensive inducers | ^1,10,21^ |
|  |  |  |  | *Applications:* Production of *p*-coumaric acid | |  |
| **NahR** | *Psal* | Naphthalene, salicylate and derivatives | 5-10 µM (naphthalene) | - Salicylate is an approved drug for humans - Characterization | - Bimodal response | ^1,22–24^ |
|  |  |  |  | *Applications:* Naphthalene biosensor; biofilm research, gene expression cascade with XylS | |  |
| **AlkS** | *PalkB* | Short-chain alkanes | 0.05-0.1% (v/v) | - Characterization | - Volatile inducers (DCPK less volatile and non-metabolizable) - Catabolite repression - Leaky expression | ^10,25^ |
| **MekR** | *PmekA* | Methyl ethyl ketone (MEK) and derivatives | 1% (v/v) (acetone) | - Well characterized - No metabolization of MEK: dose-dependent response - Tight regulation | - MEK is volatile and toxic - Catabolite repression | ^14,26^ |
|  |  |  |  | *Application:* Expression of Cre for recombineering | |  |
| **NagR** | *pNagAA* | Salicylate | 0.01–1 mM | - Salicylate is an approved drug for humans   *Applications:* Production of phenazine, phenol and 3-methylcatechol | | ^27–31^ |
| **BenR** | *Pb* | Benzoate | 0.05-1 mM | - Unimodal response - Tight regulation | - Metabolization of inducer: no longterm dose-dependent response | ^32,33^ |
|  |  |  |  | *Application:* Development of a bicistronic reporter cassette | |  |
| **ChnR** | *PchnB* | Cyclohexanone | 0.1-1 mM | - Tight regulation   *Applications:* Oxidative stress research; haloalkane degradation; regulation of biofilm formation | | ^34–36^ |
| **CprK1** | *P_DB3_* | 3-chloro-4-hydroxyphenylacetic acid | 1 mM | - Characterization | | ^18,37^ |
| **PcaU** | *PpcaU* | Protocatechuate | 0.003-10 mM | - Metabolization of inducer: no longterm dose-dependent response   *Application:* Protocatechuate biosensor | | ^38^ |
| **CatM** | *PcatB* | Muconate precursors | / | - Poor muconate uptake - Metabolization of inducer: no longterm dose-dependent response   *Application:* Muconate biosensor | | ^39^ |
| **CscR** | *PcscA* | Sucrose | / | - No metabolization of inducer in wildtype: dose-dependent response - Non-toxic inducer   *Application:* Sucrose metabolization | | ^40,41^ |
| **IvaR** | *PIvaA* | Levulinic acid | / | - Well characterized | - Metabolization of inducer: no longterm dose-dependent response | ^42,43^ |
|  |  |  |  | *Application:* Production of sclPHAs | |  |
| **MtlR** | *PmtlE* | D-mannitol, D-arabitol, D-glucitol | 0.2% (w/v) | - Well characterized - Non-toxic inducers - Tight regulation | | ^44^ |
| **GcdR** | *PgcdH* | Glutarate and derivatives | 0.01-2.5 mM | - Well characterized - Tight regulation   *Application:* Glutarate biosensor | | ^45,46^ |
| ***Negatively regulated induction systems*** | | | | | | |
| **LacI** | *Plac, PlacUV5, Ptac,*  *Ptrc* | Lactose, Isopropyl β-D-1-thiogalactopyranoside (IPTG) | 0.5-2.5 mM (IPTG) | - Extensively characterized - No metabolization of lactose or IPTG: dose-dependent response | - Toxicity of IPTG - Leaky expression - Expensive inducers | ^10,21,27,36,47–50^ |
|  |  |  |  | *Applications:* Expression of Bxb1 integrase for genomic integration; biodesulfurization; production of *p-*hydroxybenzoate, ethanol and recombinant proteins | |  |
| **TetR** | *Ptet* | anhydrotetracycline (aTc) | 100 nM | - Tight regulation - No metabolization of inducer: dose-dependent response | - Unreliable functioning (probably due to unbalanced TetR levels) - Toxicity of aTc | ^21,44,51,52^ |
|  |  |  |  | *Application:* Tubulysin production |  |  |
| **EilR** | *P_JExA1_* | Crystal violet (CV) and other cationic dyes | 1 nM (CV) | - Well characterized - Tight regulation - No metabolization of inducer: dose-dependent response - No crosstalk with *P_BAD_, Plac, Ptet* - Cheap inducers | - Toxicity of CV | ^52^ |
| **ArsR** | *Pars1, Pars2* | Arsenic salts (NaAsO_2_ or NaHAsO_4_) | / | - Metabolization of inducer: no longterm dose-dependent response - Toxicity of arsenic salts | | ^53,54^ |
| **CsiR** | *PcsiD* | Glutarate and derivatives | 0.005-0.02 mM | - Well characterized in putida - Low basal expression   *Application*: Glutarate biosensor | | ^45,46^ |
| **DntR** | *P_DNT_* | Salicylate | 10 mM | - Salicylate is approved human drug   *Application*: 24-DNT degradation | | ^34,55^ |
| ***Other induction systems*** | | | | | | |
| **PelA** | *PpelA* | Intracellular c-di-GMP | N/A | *Application*: Intracellular c-di-GMP biosensor | | ^36^ |
| **FleQ** | *PcdrA* | Intracellular c-di-GMP | N/A | *Application*: Intracellular c-di-GMP biosensor | | ^22,56^ |
| **PpuI-RsaL-PpuR QS system** |  | AHLs 3-oxo-C10 and 3-oxo-C12 | N/A | - Extensively characterized - Native putida IsoF system | | ^57–59^ |
| **CcaSR** | *P_cpcG2_* | 520 nm | N/A | - Characterization - Tight regulation - Unimodal response | - No OFF switch as in *E. coli* (not inhibited by red light) | ^60^ |
| **RoxRS QS system** | *P_Rox132_, P_Rox306_,*  *P_Rox3061_* | Tetradecanoic acid and derivatives | N/A | - Well characterized - Set of promoters for response at different cell densities - Native system   *Application:* Recombinant protein production | | ^61,62^ |
| **RhlRI QS system** | *PrhlA* | N-acyl homoserine lactones | N/A | - Poorly characterized   *Application:* Rhamnolipid production | | ^63,64^ |
| **cI857** | *P_L_* | >37°C | N/A | - Characterization - Tight regulation | - Need for a heat-tolerant strain - Maximum 3h of induction | ^65,66^ |
|  |  |  |  | *Application:* Recombinant protein production; inhibition of mismatch repair for recombineering | |  |
| ***Alternative polymerases*** | | | | | | |
| **T5 RNAP** | *P_T5_* | LacI-IPTG | 1 mM (IPTG) | - Drawbacks of LacI system   *Application:* Expression of His_6_-tagged recombinant proteins | | ^67^ |
| **T7 RNAP** | *P_T7_* | LacI-IPTG or  XylS-*m*-toluate | 10 mM (IPTG) | - Characterization - Orthogonality - High expression levels | - Toxicity of T7 RNAP - Drawbacks of LacI system | ^10,68,69^ |
|  |  |  |  | *Application:* Production of *p*-coumaric acid and recombinant proteins | |  |

1. Galvão, T. C. & De Lorenzo, V. Transcriptional regulators à la carte: Engineering new effector specificities in bacterial regulatory proteins. *Curr. Opin. Biotechnol.* **17**, 34–42 (2006).

2. Silva-Rocha, R., de Jong, H., Tamames, J. & de Lorenzo, V. The logic layout of the TOL network of Pseudomonas putida pWW0 plasmid stems from a metabolic amplifier motif (MAM) that optimizes biodegradation of m-xylene. *BMC Syst. Biol.* **5**, (2011).

3. Guantes, R., Benedetti, I., Silva-Rocha, R. & De Lorenzo, V. Transcription factor levels enable metabolic diversification of single cells of environmental bacteria. *ISME J.* **10**, 1122–1133 (2016).

4. Silva-Rocha, R. & de Lorenzo, V. Stochasticity of TOL plasmid catabolic promoters sets a bimodal expression regime in Pseudomonas putida mt-2 exposed to m-xylene. *Mol. Microbiol.* **86**, 199–211 (2012).

5. De Las Heras, A. & De Lorenzo, V. In situ detection of aromatic compounds with biosensor Pseudomonas putida cells preserved and delivered to soil in water-soluble gelatin capsules. *Anal. Bioanal. Chem.* **400**, 1093–1104 (2011).

6. De Las Heras, A., Carreño, C. A. & De Lorenzo, V. Stable implantation of orthogonal sensor circuits in Gram-negative bacteria for environmental release. *Environ. Microbiol.* **10**, 3305–3316 (2008).

7. Silva-Rocha, R. & De Lorenzo, V. The TOL network of Pseudomonas putida mt-2 processes multiple environmental inputs into a narrow response space. *Environ. Microbiol.* **15**, 271–286 (2013).

8. Volke, D. C., Friis, L., Wirth, N. T., Turlin, J. & Nikel, P. I. Synthetic control of plasmid replication enables target- and self-curing of vectors and expedites genome engineering of Pseudomonas putida. *Metab. Eng. Commun.* **10**, e00126 (2020).

9. Dammeyer, T. *et al.* Efficient production of soluble recombinant single chain Fv fragments by a Pseudomonas putida strain KT2440 cell factory. *Microb. Cell Fact.* **10**, (2011).

10. Calero, P., Jensen, S. I. & Nielsen, A. T. Broad-Host-Range ProUSER Vectors Enable Fast Characterization of Inducible Promoters and Optimization of p-Coumaric Acid Production in Pseudomonas putida KT2440. *ACS Synth. Biol.* **5**, 741–753 (2016).

11. Gawin, A., Valla, S. & Brautaset, T. The XylS/Pm regulator/promoter system and its use in fundamental studies of bacterial gene expression, recombinant protein production and metabolic engineering. *Microb. Biotechnol.* **10**, 702–718 (2017).

12. Goñi-Moreno, Á., Benedetti, I., Kim, J. & De Lorenzo, V. Deconvolution of Gene Expression Noise into Spatial Dynamics of Transcription Factor-Promoter Interplay. *ACS Synth. Biol.* **6**, 1359–1369 (2017).

13. Martínez-García, E. & de Lorenzo, V. Engineering multiple genomic deletions in Gram-negative bacteria: Analysis of the multi-resistant antibiotic profile of Pseudomonas putida KT2440. *Environ. Microbiol.* **13**, 2702–2716 (2011).

14. Luo, X. *et al.* Pseudomonas putida KT2440 markerless gene deletion using a combination of λ Red recombineering and Cre/loxP site-specific recombination. *FEMS Microbiol. Lett.* **363**, 1–7 (2016).

15. Wirth, N. T., Kozaeva, E. & Nikel, P. I. Accelerated genome engineering of Pseudomonas putida by I-SceI―mediated recombination and CRISPR-Cas9 counterselection. *Microb. Biotechnol.* (2019). doi:10.1111/1751-7915.13396

16. Martínez, V., García, P., García, J. L. & Prieto, M. A. Controlled autolysis facilitates the polyhydroxyalkanoate recovery in Pseudomonas putida KT2440. *Microb. Biotechnol.* **4**, 533–547 (2011).

17. Borrero-de Acuña, J. M., Hidalgo-Dumont, C., Pacheco, N., Cabrera, A. & Poblete-Castro, I. A novel programmable lysozyme-based lysis system in Pseudomonas putida for biopolymer production. *Sci. Rep.* **7**, 1–11 (2017).

18. Batianis, C. *et al.* An expanded CRISPRi toolbox for tunable control of gene expression in Pseudomonas putida. *Microb. Biotechnol.* **13**, 368–385 (2020).

19. Jeske, M. & Altenbuchner, J. The Escherichia coli rhamnose promoter rhaPBAD is in Pseudomonas putida KT2440 independent of Crp-cAMP activation. *Appl. Microbiol. Biotechnol.* **85**, 1923–1933 (2010).

20. Kim, S. K. *et al.* CRISPR interference-mediated gene regulation in Pseudomonas putida KT2440. *Microb. Biotechnol.* (2019). doi:10.1111/1751-7915.13382

21. Cook, T. B. *et al.* Genetic tools for reliable gene expression and recombineering in Pseudomonas putida. *J. Ind. Microbiol. Biotechnol.* **45**, 517–527 (2018).

22. Jimenez-Fernandez, A., Lopez-Sanchez, A., Calero, P. & Govantes, F. The c-di-GMP phosphodiesterase BifA regulates biofilm development in Pseudomonas putida. *Environ. Microbiol. Rep.* **7**, 78–84 (2015).

23. Werlen, C., Jaspers, M. C. M. & Van Der Meer, J. R. Measurement of Biologically Available Naphthalene in Gas and Aqueous Phases by Use of a Pseudomonas putida Biosensor. *Appl. Environ. Microbiol.* **70**, 43–51 (2004).

24. Becker, P. D., Royo, J. L. & Guzman, C. A. Based on the salicylate-dependent control circuit encompassing nahR/P sal::xylS2 for biotechnological applications. *Bioeng. Bugs* **1**, 244–251 (2010).

25. Rojo, F. Degradation of alkanes by bacteria: Minireview. *Environ. Microbiol.* **11**, 2477–2490 (2009).

26. Graf, N. & Altenbuchner, J. Functional characterization and application of a tightly regulated MekR/P mekA expression system in Escherichia coli and Pseudomonas putida. *Appl. Microbiol. Biotechnol.* **97**, 8239–8251 (2013).

27. Verhoef, S., Ballerstedt, H., Volkers, R. J. M., De Winde, J. H. & Ruijssenaars, H. J. Comparative transcriptomics and proteomics of p-hydroxybenzoate producing Pseudomonas putida S12: Novel responses and implications for strain improvement. *Appl. Microbiol. Biotechnol.* **87**, 679–690 (2010).

28. Askitosari, T. D., Boto, S. T., Blank, L. M. & Rosenbaum, M. A. Boosting heterologous phenazine production in pseudomonas putida KT2440 through the exploration of the natural sequence space. *Front. Microbiol.* **10**, 1–12 (2019).

29. Schmitz, S., Nies, S., Wierckx, N., Blank, L. M. & Rosenbaum, M. A. Engineering mediator-based electroactivity in the obligate aerobic bacterium Pseudomonas putida KT2440. *Front. Microbiol.* **6**, 1–13 (2015).

30. Wierckx, N. J. P., Ballerstedt, H., Bont, J. a M. De & Wery, J. Engineering of Solvent-Tolerant Pseudomonas putida S12 for Bioproduction of Phenol from Glucose. *Appl. Environ. Microbiol.* **71**, 8221–8227 (2005).

31. Hüsken, L. E., Beeftink, R., De Bont, J. A. M. & Wery, J. High-rate 3-methylcatechol production in Pseudomonas putida strains by means of a novel expression system. *Appl. Microbiol. Biotechnol.* **55**, 571–577 (2001).

32. Silva-Rocha, R. & de Lorenzo, V. A GFP-lacz bicistronic reporter system for promoter analysis in environmental gram-negative bacteria. *PLoS One* **7**, (2012).

33. Benedetti, I. M., de Lorenzo, V. & Silva-Rocha, R. Quantitative, Non-Disruptive Monitoring of Transcription in Single Cells with a Broad-Host Range GFP-luxCDABE Dual Reporter System. *PLoS One* **7**, (2012).

34. Akkaya, Ö., Pérez-pantoja, D. R., Calles, B., Nikel, P. I. & de Lorenzo, V. The Metabolic Redox Regime of Pseudomonas putida Tunes Its Evolvability toward Novel Xenobiotic Substrates. *MBio* **9**, 1–16 (2018).

35. Benedetti, I., Nikel, P. I. & de Lorenzo, V. Data on the standardization of a cyclohexanone-responsive expression system for Gram-negative bacteria. *Data Br.* **6**, 738–744 (2016).

36. Benedetti, I., de Lorenzo, V. & Nikel, P. I. Genetic programming of catalytic Pseudomonas putida biofilms for boosting biodegradation of haloalkanes. *Metab. Eng.* **33**, 109–118 (2016).

37. Kemp, L. R., Dunstan, M. S., Fisher, K., Warwicker, J. & Leys, D. The transcriptional regulator CprK detects chlorination by combining direct and indirect readout mechanisms. *Philos. Trans. R. Soc. B Biol. Sci.* **368**, (2013).

38. Jha, R. K. *et al.* A protocatechuate biosensor for Pseudomonas putida KT2440 via promoter and protein evolution. *Metab. Eng. Commun.* **6**, 33–38 (2018).

39. Bentley, G. J. *et al.* Engineering glucose metabolism for enhanced muconic acid production in Pseudomonas putida KT2440. *Metab. Eng.* **59**, 64–75 (2020).

40. Fedeson, D. T., Saake, P., Calero, P., Nikel, P. I. & Ducat, D. C. Biotransformation of 2,4-dinitrotoluene in a phototrophic co-culture of engineered Synechococcus elongatus and Pseudomonas putida. *Microb. Biotechnol.* (2020). doi:10.1111/1751-7915.13544

41. Löwe, H., Sinner, P., Kremling, A. & Pflüger-Grau, K. Engineering sucrose metabolism in Pseudomonas putida highlights the importance of porins. *Microb. Biotechnol.* **13**, 97–106 (2018).

42. Cha, D., Ha, H. S. & Lee, S. kuk. Metabolic engineering of Pseudomonas putida for the production of various types of short-chain-length polyhydroxyalkanoates from levulinic acid. *Bioresour. Technol.* **309**, 123332 (2020).

43. Rand, J. M. *et al.* A metabolic pathway for catabolizing levulinic acid in bacteria. *Nat. Microbiol.* **2**, 1624–1634 (2017).

44. Hoffmann, J. & Altenbuchner, J. Functional characterization of the mannitol promoter of Pseudomonas fluorescens DSM 50106 and its application for a mannitol-inducible expression system for Pseudomonas putida KT2440. *PLoS One* **10**, 1–22 (2015).

45. Thompson, M. G. *et al.* Robust Characterization of Two Distinct Glutarate Sensing Transcription Factors of Pseudomonas putida l -Lysine Metabolism. *ACS Synth. Biol.* **8**, 2385–2396 (2019).

46. Zhang, M. *et al.* Regulation of Glutarate Catabolism by GntR Family Regulator CsiR and LysR Family Regulator GcdR in Pseudomonas putida KT2440. *MBio* **10**, 1–15 (2019).

47. Elmore, J. R., Furches, A., Wolff, G. N., Gorday, K. & Guss, A. M. Development of a high efficiency integration system and promoter library for rapid modification of Pseudomonas putida KT2440. *Metab. Eng. Commun.* **5**, 1–8 (2017).

48. Lieder, S., Nikel, P. I., de Lorenzo, V. & Takors, R. Genome reduction boosts heterologous gene expression in Pseudomonas putida. *Microb. Cell Fact.* **14**, 1–14 (2015).

49. Nikel, P. I. & de Lorenzo, V. Robustness of Pseudomonas putida KT2440 as a host for ethanol biosynthesis. *N. Biotechnol.* **31**, 562–571 (2014).

50. Martínez, I., Mohamed, M. E. S., Rozas, D., García, J. L. & Díaz, E. Engineering synthetic bacterial consortia for enhanced desulfurization and revalorization of oil sulfur compounds. *Metab. Eng.* **35**, 46–54 (2016).

51. Chai, Y. *et al.* Heterologous expression and genetic engineering of the tubulysin biosynthetic gene cluster using red/ET recombineering and inactivation mutagenesis. *Chem. Biol.* **19**, 361–371 (2012).

52. Ruegg, T. L. *et al.* Jungle Express is a versatile repressor system for tight transcriptional control. *Nat. Commun.* **9**, 1–13 (2018).

53. Páez-Espino, A. D., Durante-Rodríguez, G. & de Lorenzo, V. Functional coexistence of twin arsenic resistance systems in Pseudomonas putida KT2440. *Environ. Microbiol.* **17**, 229–238 (2015).

54. Páez-Espino, A. D., Nikel, P. I., Chavarría, M. & de Lorenzo, V. ArsH protects Pseudomonas putida from oxidative damage caused by exposure to arsenic. *Environ. Microbiol.* **00**, 1–13 (2020).

55. de las Heras, A., Chavarría, M. & de Lorenzo, V. Association of dnt genes of Burkholderia sp. DNT with the substrate-blind regulator DntR draws the evolutionary itinerary of 2,4-dinitrotoluene biodegradation. *Mol. Microbiol.* **82**, 287–299 (2011).

56. Rybtke, M. T. *et al.* Fluorescence-based reporter for gauging cyclic Di-GMP levels in Pseudomonas aeruginosa. *Appl. Environ. Microbiol.* **78**, 5060–5069 (2012).

57. Cárcamo-Oyarce, G., Lumjiaktase, P., Kümmerli, R. & Eberl, L. Quorum sensing triggers the stochastic escape of individual cells from Pseudomonas putida biofilms. *Nat. Commun.* **6**, 1–9 (2015).

58. Dubern, J. F., Lugtenberg, B. J. J. & Bloemberg, G. V. The ppuI-rsaL-ppuR quorum-sensing system regulates biofilm formation of Pseudomonas putida PCL1445 by controlling biosynthesis of the cyclic lipopeptides putisolvins I and II. *J. Bacteriol.* **188**, 2898–2906 (2006).

59. Fekete, A. *et al.* Dynamic regulation of N-acyl-homoserine lactone production and degradation in Pseudomonas putida IsoF. *FEMS Microbiol. Ecol.* **72**, 22–34 (2010).

60. Hueso-Gil, A., Nyerges, Á., Pál, C., Calles, B. & De Lorenzo, V. Multiple-Site Diversification of Regulatory Sequences Enables Interspecies Operability of Genetic Devices. *ACS Synth. Biol.* **9**, 104–114 (2020).

61. Meyers, A., Furtmann, C., Gesing, K., Tozakidis, I. E. P. & Jose, J. Cell density-dependent auto-inducible promoters for expression of recombinant proteins in Pseudomonas putida. *Microb. Biotechnol.* (2019). doi:10.1111/1751-7915.13455

62. Espinosa-urgel, M. & Ramos, J. Cell Density-Dependent Gene Contributes to Efficient Seed Colonization by Pseudomonas putida KT2440. *Appl. Environ. Microbiol.* **70**, 5190–5198 (2004).

63. Cao, L. *et al.* Construction of a stable genetically engineered rhamnolipid-producing microorganism for remediation of pyrene-contaminated soil. *World J. Microbiol. Biotechnol.* **28**, 2783–2790 (2012).

64. Cha, M., Lee, N., Kim, M., Kim, M. & Lee, S. Heterologous production of Pseudomonas aeruginosa EMS1 biosurfactant in Pseudomonas putida. *Bioresour. Technol.* **99**, 2192–2199 (2008).

65. Aparicio, T., de Lorenzo, V. & Martínez-García, E. Improved Thermotolerance of Genome-Reduced Pseudomonas putida EM42 Enables Effective Functioning of the P L /cI857 System. *Biotechnol. J.* **14**, 1–8 (2019).

66. Aparicio, T. *et al.* Mismatch repair hierarchy of Pseudomonas putida revealed by mutagenic ssDNA recombineering of the pyrF gene. *Environ. Microbiol.* **00**, (2019).

67. Bertani, I., Devescovi, G. & Venturi, V. Controlled specific expression and purification of 6ÃHis-tagged proteins in Pseudomonas . *FEMS Microbiol. Lett.* **179**, 101–106 (1999).

68. Herrero, M., Lorenzo, V. de, Ensley, B. & Timmis, K. N. A T7 RNA polymerase-based system for the construction of Pseudomonas strains with phenotypes dependent on TOL-meta pathway effectors. *Gene* **134**, 103–106 (1993).

69. Troeschel, S. C. *et al.* Novel broad host range shuttle vectors for expression in Escherichia coli, Bacillus subtilis and Pseudomonas putida. *J. Biotechnol.* **161**, 71–79 (2012).
